# BVDV NS5A Binds to CKAP2 and Activates the PI3K/AKT/mTOR Pathway to Facilitate Virus Transmission Through Tunneling Nanotubes

**DOI:** 10.3390/vetsci13060505

**Published:** 2026-05-22

**Authors:** Jiying Yin, Yanan Zhu, Jiating Zhang, Zehui Zhou, Ning He, Hongming Zhou, Xiaoqun Liu, Yixing Zhao, Longge Zhao, Ying Zong, Naichao Diao, Kun Shi, Nan Li, Rui Du

**Affiliations:** 1 College of Animal Science and Technology, Jilin Agricultural University, Changchun 130118, China; yinjiying1103@163.com (J.Y.); 18729193031@163.com (Y.Z.); z2323134317@163.com (J.Z.); zehui.zhou0424@gmail.com (Z.Z.); 18043212558@163.com (N.H.); xm757137@icloud.com (H.Z.); 15844350065@163.com (X.L.); yixing1228@163.com (Y.Z.); zhaolg0502@163.com (L.Z.); 2 College of Chinese Medicinal Materials, Jilin Agricultural University, Changchun 130118, China; zongying@jlau.edu.cn (Y.Z.); diaonaichao@jlau.edu.cn (N.D.); 3 Institute of Animal Husbandry and Veterinary Medicine, Changchun Academy of Agricultural Sciences, Changchun 130062, China; 4 Laboratory of Production and Product Application of Sika Deer of Jilin Province, Jilin Agricultural University, Changchun 130118, China; 5 Key Laboratory of Animal Production, Product Quality and Security, Ministry of Education, Jilin Agricultural University, Changchun 130118, China

**Keywords:** bovine viral diarrhea virus, tunneling nanotubes, CKAP2, NS5A, PI3K/AKT/mTOR

## Abstract

Bovine viral diarrhea virus (BVDV), a significant global pathogen threatening cattle industries worldwide, presents substantial challenges for disease control. Its ability to infect cattle across all age groups, coupled with incompletely understood transmission mechanisms, complicates prevention and treatment strategies. We previously reported that BVDV induced tunneling nanotubes (TNTs)—F-actin-rich cytoplasmic connections between adjacent cells—and utilizes these structures for intercellular transmission. In this study, we used lentiviral transfection to express various structural and non-structural proteins of BVDV and identified NS5A as a critical viral protein that induces the formation of TNTs. RNA-seq analysis revealed that CKAP2, a host protein, plays a key role in TNT generation, with the PI3K/AKT/mTOR signaling pathway being essential for this process. Further investigation demonstrated that CKAP2 interacts with BVDV NS5A, triggering the activation of the PI3K/AKT/mTOR pathway, thereby promoting TNT formation and enhancing viral dissemination. Our data highlight a previously unknown mechanism of BVDV spreading and replication, which could have significant implications for within-host spread and immune evasion.

## Simple Summary

Bovine viral diarrhea virus (BVDV) is a serious disease that affects cattle worldwide, leading to significant financial losses for the livestock industry. We know that the virus is dangerous, but we do not fully understand all the ways in which it spreads while remaining hidden from the animal’s immune system. In this study, we investigated how the virus creates tiny bridge-like connections called tunneling nanotubes (TNTs) to move directly from one cell to another. We discovered that a specific part of the virus binds to a protein inside the cow’s cells. This interaction triggers a biological “construction signal” that forces the cell to build these bridges. Once formed, these tubes allow the virus to travel safely to neighboring cells, avoiding the immune system’s defenses. Our research demonstrates that by blocking this specific host protein or the internal signaling pathway it activates, we can significantly reduce the formation of these bridges and hinder the virus’s ability to multiply. These findings are valuable to society because they reveal a new target for developing treatments or vaccines that protect animal health and ensure a more stable global food supply.

## 1. Introduction

Bovine viral diarrhea virus (BVDV), a member of the Flaviviridae family and Pestivirus genus, is an enveloped virus measuring 40–60 nm in diameter [1]. It infects various mammals, particularly cattle, causing severe production losses due to clinical manifestations such as diarrhea, respiratory distress, reproductive disorders, and immunosuppression [2]. These effects pose a major threat to global livestock industries, leading to significant economic impacts. BVDV evades host immune defenses and can be vertically transmitted across the placenta, resulting in persistent infections in offspring [3]. A key factor in viral persistence is its ability to spread via direct cell-to-cell transmission, which serves as the primary mode of infection [4,5]. Understanding the mechanisms underlying this transmission route and its contribution to persistent infection remains a critical research priority in the field.

In recent years, tunnel nanotubes (TNTs) have been recognized as important conduits for intercellular viral transmission [6]. However, whether BVDV utilizes this pathway remains unreported. TNTs, first identified in 2004, are F-actin-rich, filamentous tubular structures that create cytoplasmic connections between neighboring or distant cells [7]. In particular, F-actin polymerisation is crucial for the formation and stability of TNTs. These dynamic structures have been extensively documented in recent studies as a viral transmission route that can mediate pathogen transfer during their early formation stages. TNT-mediated transport offers viruses significant advantages, particularly in shielding viral components from the extracellular environment. For instance, bovine herpesvirus type 1 (BoHV-1) has been shown to exploit TNT formation for efficient transfer between epithelial cells and fibroblasts [8]. Notably, viral transmission via TNTs occurs at markedly higher speeds compared to extracellular transfer, indicating that this direct cell-to-cell mechanism represents a more efficient host infection strategy than extracellular dissemination. Moreover, viral exploitation of TNTs for intercellular delivery significantly enhances their ability to evade host immune defenses. For example, studies on human T cells leukemia virus type 1 (HTLV-1) demonstrate that TNT-mediated viral transfer allows the pathogen to bypass immune surveillance, shedding light on novel immune evasion strategies [9]. Similarly, in HIV-infected individuals, the extensive TNT networks connecting T cells facilitate direct cell-to-cell transmission, enabling the virus to efficiently infect neighboring cells even in the presence of neutralizing antibodies [10]. Further evidence suggested that Ebola virus utilizes TNTs as an alternative transmission route to avoid immune detection, revealing a previously unrecognized mode of viral dissemination within the host [11]. Additionally, Anna Pepe et al. revealed that SARS-CoV-2 exploits TNTs to infect brain cells—a critical finding given that neuronal cells lack ACE2 receptors, the virus’s primary entry point. By hijacking TNTs, SARS-CoV-2 can spread from susceptible cells to the brain, accelerating infection while evading immune responses [12]. These findings collectively uncover novel mechanisms of viral immune evasion and intercellular transmission.

Our research group’s previous studies have found that: Tunneling nanotubes provide a new route for bovine viral diarrhea virus spreading [13]. We contend that studying the mechanisms of BVDV transmission between target cells will likely lead to effective strategies for the prevention and treatment of BVDV infections in the future.

## 2. Materials and Methods

### 2.1. Cells and Virus

Madin-Darby Bovine Kidney cells (MDBK; Cell Bank Type Culture Collection of the Chinese Academy of Sciences, Shanghai, China) were obtained and maintained in Dulbecco’s modified Eagle’s medium (DMEM; Gibco, Grand Island, NY, USA) containing 10% fetal bovine serum (FBS; Gibco, Grand Island, NY, USA), under standard culture conditions at 37 °C with 5% CO_2_ in a humidified atmosphere. The BVDV strain NADL (cytopathic biotype, genotype 1a) was obtained from the American Type Culture Collection (ATCC, Manassas, VA, USA). The following cell lines were constructed in our laboratory: MDBK-CKAP2-knockout (MDBK CKAP2 KO), MDBK-CKAP2-KO + CKAP2 rescue (MDBK CKAP2 KO + CKAP2), and MDBK-CKAP2-overexpression (OE MDBK CKAP2).

### 2.2. Antibodies and Reagents

In this study, we used Bovine Viral Diarrhea Virus Type 1&2 (BVDV-1&2) MAb E2 gp53 (#348, Veterinary Medical Research & Development, Pullman, WA, USA); TRITC Phalloidin (40734ES75, YEASEN, Shanghai, China); and goat anti-mouse IgG(H + L)-FITC-conjugated (#ab6785, Abcam, Cambridge, UK). We used the antibodies anti-mTOR (66888–1-Ig, Proteintech, Wuhan, Hubei, China), anti-PI3K (67071–1-Ig, Proteintech, Wuhan, China), and anti-AKT (60203–2-Ig, Proteintech, Wuhan, China); anti-p-mTOR (bs-3494R, Bioss, Beijing, China), anti-p-PI3K (bs-5507R, Bioss, Beijing, China), anti-p-AKT (bs-0876R, Bioss, Beijing, China), and anti-F-actin (bs-1571R, Bioss, Beijing, China); horseradish peroxidase (HRP)-conjugated goat anti-mouse IgG (As003, ABclonal, Wuhan, China) and goat anti-rabbit IgG (As014, ABclonal, Wuhan, China); and HRP-conjugated goat anti-bovine IgG (H + L) (#5220-0361, SeraCare, Milford, MA, USA). Furthermore, we used the AEC Peroxidase Substrate Kit 20× (A2010, Solarbio, Beijing, China); Ly294002 (HY-10108, MCE, Monmouth Junction, NJ, USA); Perifosine (HY-50909, MCE, Monmouth Junction, NJ, USA); and Rapamycin (HY-10219, MCE, Monmouth Junction, NJ, USA).

### 2.3. Immunofluorescence

MDBK cells were plated onto glass coverslips in 48-well plates at a density of 2 × 10^5^ cells per well and infected at a multiplicity of infection (MOI) of 1. Following infection, cells were thoroughly washed at designated time points and fixed with 4% paraformaldehyde (PFA) for 10 min, then permeabilized with 0.5% Triton X-100 in PBS for 8 min. After blocking with 3% BSA, the samples were incubated overnight with an anti-BVDV E2 monoclonal antibody (1:100, #348, Veterinary Medical Research & Development, Pullman, WA, USA). This was followed by a 2 h incubation period with a goat anti-mouse IgG (H&L)-FITC secondary antibody (1:40, #ab6785, Abcam, Cambridge, UK). Nuclei were counterstained with DAPI (4′,6-diamidino-2-phenylindole), and coverslips were mounted on glass slides using FluoroGuard™ antifade reagent (P36982, invitrogen, Waltham, MA, USA). Imaging was performed using a Leica STELLARIS 5 confocal microscope (Leica Microsystems CMS GmbH, Mannheim, Germany).

### 2.4. Total Cellular RNA Extraction and Quantitative Real-Time PCR

MDBK cells were cultured in 75 cm^2^ cell culture flasks (#430720U, Corning, NY, USA). Total cellular RNA was extracted as follows: Briefly, 500 µL of TRIzol Reagent was added to the cultured cells and blown repeatedly to fully lyse the cells. Then, 500 µL of TRIzol Pal^TM^ was added, shaken vigorously for 15 s, and centrifuged at 12,000 r/min at 4 °C for 10 min. The upper colorless aqueous phase was aspirated, and its volume was measured. An equal volume of 70% ethanol was added, inverted, and mixed. The mixture was then processed with an RNA purification column: 700 µL of RW1 was added and centrifuged for 30 s; 500 µL of RW2 was added and centrifuged for 30 s; and finally, 35 µL of RNase-Free water was added and centrifuged for 2 min, and the supernatant was collected to obtain the RNA. The 5′ UTR of BVDV is highly conserved and is one of the key markers for detecting BVDV replication. The BVDV 5′UTR primer sequences are as follows: forward primer, 5′-AGTCGTCARTGGTTCGAC-3′; reverse primer, 5′-TCAACTCCATGTGCCATGTAC-3′. All primers were synthesized by Sangon Biotech (Shanghai, China). RT-qPCR was performed using the TB Green^®^ Premix Ex Taq™ Kit (RR820, Takara, Shanghai, China). Each 25 μL reaction mixture contained 12.5 μL of TB Green Premix Ex Taq™, 1 μL each of forward and reverse primers (10 μM), 1 μL of diluted cDNA template, and 9.5 μL of nuclease-free water. The thermal cycling conditions were as follows: 30 s at 95 °C, 5 s at 95 °C, and 30 s at 60 °C, for a total of 35 cycles. The relative expression of the 5′UTR transcript was calculated using the comparative Ct method (2^−ΔΔCt^), with normalization to the GAPDH. All samples were run in triplicate, and the data are presented as the mean ± standard deviation (SD).

### 2.5. Western Blotting

Total protein was extracted with cell lysis buffer, and an equal amount of protein was electrophoresed at a constant voltage of 80 V. When the protein samples passed through the concentration gel, a constant voltage of 120 V was used until the end of electrophoresis, and then the samples shifted to polyvinylidene fluoride (PVDF) membranes. After sealing with 5% BSA solution for 2 h, the membranes were rinsed in Tris-buffered saline (TBS) including 0.1%Tween-20 (TBS-T) for 10 min 3 times. Anti-F-actin (1:500 dilution), anti-mTOR (1:1000 dilution), anti-Akt (1:5000 dilution), anti-PI3K (1:2000 dilution), anti-p-mTOR (1:2000 dilution), anti-p-AKT (1:1000 dilution), and anti-p-PI3K (1:1000 dilution) were used as primary antibodies and incubated overnight at 4 °C; the secondary antibody conjugated with horseradish peroxidase (HRP) was added in a dilution of 1:5000 for 2 h at room temperature, and then the immunoreactive protein bands were visualized. We used an emitter-coupled logic (ECL) substrate (#180-5001, Tanon, Shanghai, China) to react with the ERP coupling of the secondary antibody to observe the protein–antibody complexes using a chemiluminescence imager (MiniChemi610, SageCreation, Beijing, China). Finally, Image-Pro plus 6.0 software (Media Cybernetics, Rockville, MD, USA) was used to analyze the signal intensity of the protein bands.

### 2.6. Lentivirus Packaged Viral Proteins and Transfection of Cells

The full-length coding sequences of eleven BVDV strain NADL genes, including Npro, C, Erns, E1, E2, NS2, NS4A, NS4B, NS5A, NS5B, and NS3, were amplified by PCR using primers designed according to the published sequence in GenBank. All primers were synthesized by Sangon Biotech (Shanghai, China). The amplified fragments were digested with the corresponding restriction enzymes and ligated into the pLenti-EF1a-EGFP-P2A-Puro-CMV-MCS-3Flag lentiviral vector that had been linearized with the same enzymes. The ligation products were transformed into competent *E. coli* (DH5α), and positive clones were selected on LB agar plates containing ampicillin. Plasmids were extracted and verified by DNA sequencing. The target gene plasmid, packaging plasmid psPAX2, and envelope plasmid pMD2.G were mixed at a 3:2:1 ratio; Opti-MEM (Gibco, Grand Island, NY, USA) was used to obtain a final volume of 250 µL; and the mixture was incubated at room temperature for 10 min. At the same time, 34.5 µL of PEI (Servicebio, Wuhan, China) was mixed with Opti-MEM to reach a total volume of 250 μL and vortexed, and then the mixture was allowed to stand at room temperature for 5 min. Then, we slowly added the PEI solution to the plasmid mixture, mixed gently, and placed in an incubator at room temperature for 15 min to form the transfection complex. The complex was added dropwise to the cell culture system, gently mixed, and then placed in an incubator for continued culture. At 18 h post-transfection, the medium was replaced with fresh complete medium. At 48 h post-transfection, the culture supernatants were collected, centrifuged to remove cell debris, filtered through a 0.45 μm filter, aliquoted, and stored at −80 °C for future use.

### 2.7. shRNA Design and Vector Construction

A short hairpin RNA (shRNA) targeting the NS5A gene of bovine viral diarrhea virus (BVDV) was designed based on the target sequence 5′-GGTTCAATGGTGCATACTTAG-3′. Complementary DNA oligonucleotide pairs were synthesized (GenePharma, Shanghai, China) with complementary DNA oligonucleotide pairs, each comprising a sense strand (5′-GGTTCAATGGTGCATACTTAG-3′), a loop sequence (TTCAAGAGA), an antisense strand (5′-CTAAGTATGCACCATTGAACC-3′), and sequences for cloning into the pGPU6/Neo vector. The two oligonucleotides were placed in annealing buffer (10 mM Tris-HCl, 50 mM NaCl, and 1 mM EDTA, pH 8.0), heated at 95 °C for 5 min and then gradually cooled to room temperature to form double-stranded DNA. The pGPU6/Neo vector (GenePharma, Shanghai, China) was linearized with the restriction endonuclease [BbsI] and incubated overnight at 16 °C with the annealed shRNA insert fragment, followed by ligation using T4 DNA ligase. The resulting construct was then transformed into *E. coli* [DH5α] competent cells. Finally, positive colonies were selected, DNA was extracted, and the inserted shRNA sequence was verified via Sanger sequencing.

### 2.8. RNA Sequencing and Analysis

BVDV-infected MDBK cells and cytochalasin D-treated cells were collected and infected with BVDV at a multiplicity of infection (MOI) of 1 for 24 h. After RNA extraction, purification, and library construction, the samples were subjected to double-end (paired-end, PE) sequencing using Next-Generation Sequencing (NGS) on the Illumina Sequencing Platform, and junctions and low-quality sequences were removed to obtain clean reads. The libraries were subjected to paired-end (PE) sequencing based on the Illumina sequencing platform, the junctions and low-quality sequences were removed to obtain clean reads, and the expression levels of the genes were normalized to eliminate the effects of different gene lengths and sequencing differences. Differentially expressed genes (DEGs) were selected with a threshold false discovery rate (FDR) of <0.05 and an absolute value of log2 > 1 for fold change. DEGs were annotated using Gene Ontology (GO) functional enrichment analysis and the Kyoto Encyclopedia of Genes and Genomes (KEGG) pathway analysis.

### 2.9. Co-Immunoprecipitation

For protein complex extraction, 500 μL of lysis buffer was added to each vial of cells to fully lyse the sample. The lysate was centrifuged at 12,000× *g* for 5 min at 4 °C, and the supernatant was transferred to pre-cooled centrifuge tubes. Then, 20 μL of magnetic beads was added to each tube before incubating overnight on a rotary mixer at 4 °C. The samples were placed on a magnetic separation rack for 10 s, and the supernatant was discarded. Subsequently, 500 μL of lysis/wash buffer containing inhibitors was added, and the beads were gently resuspended by pipetting, followed by incubation on a rotary mixer for 10 s at room temperature. After placing the samples on the magnetic rack for another 10 s, the supernatant was discarded. Next, 200 μL of HA peptide eluent was added, mixed by pipetting, and incubated on a rotary mixer for 60 min at room temperature. Finally, the samples were placed on the magnetic rack for 10 s, and the supernatant was collected as the extracted protein complex.

### 2.10. Immunoperoxidase Monolayer Assay

Four cell lines (MDBK, MDBK CKAP2 KO, MDBK CKAP2 KO + CKAP2, and OE MDBK CKAP2) were seeded into 96-well plates at 2.2 × 10^5^ cells/mL. When cells reached 70–80% confluence, they were infected with 10-fold serial dilutions (10^−1^ to 10^−10^) of BVDV NADL virus for 1 h. For each dilution, 100 μL of the diluted virus was added to all eight wells of one column (e.g., columns 1–10 for dilutions 10^−1^–10^−10^, respectively). The last column (column 12) served as a blank control, receiving only complete medium without virus. During this period, the virus solution was gently mixed every 15 min to promote full adsorption of the virus particles to the cell surface, and incubation was completed by replacing it with complete medium with 3.5% horse serum. The cells were further incubated for 4–5 days to determine viral titer. After the indicated treatments, the culture medium was discarded, and the cells were washed three times with PBS for 5 min each time. Subsequently, the cells were fixed with 80% acetone at 4 °C for 15 min, followed by three washes with PBS for 5 min each. The cells were then treated with 3% hydrogen peroxide (H_2_O_2_) in the dark for 30 min to quench endogenous peroxidase activity. After washing three times with PBS, the cells were permeabilized with 2% Triton X-100 at 37 °C for 1 h and washed again three times with PBS for 5 min each. Next, the cells were blocked with 5% horse serum at 37 °C for 1 h, followed by three washes with PBS. The primary antibody (BVDV-positive serum) was diluted to a ratio of 1:150 in antibody diluent (1% Tween-80 and 5% horse serum) and applied to the cells for 2 h at 37 °C. After three washes with PBS, the secondary antibody (HRP-conjugated goat anti-bovine IgG (H + L)) was diluted to a ratio of 1:1000 in the same diluent and incubated for 2 h at 37 °C. The cells were then washed three times with PBS. For color development, AEC substrate solution was freshly prepared by mixing 5% solution A, 5% solution B, 5% solution C, and 85% double-distilled water (ddH_2_O). The substrate was added to the 96-well plate and incubated for 30 min. The reaction was stopped by rinsing with ddH_2_O. Stained cells were examined under a light microscope, and viral titers were calculated using the Kärber method.

### 2.11. Statistical Analysis

All data are expressed as the mean ± standard deviation (mean ± S.D.), which was determined using a one-way analysis of variance (ANOVA) and Bonferroni’s post hoc test. The statistical data are shown as graphs generated using GraphPad Prism 8.0.2 software (GraphPad Software Inc., San Diego, CA, USA). In the figures, * represents *p* < 0.05, ** represents *p* < 0.01, and *** represents *p* < 0.001.

## 3. Results

### 3.1. BVDV Promotes TNT Production by Activating the PI3K/AKT/mTOR Pathway

**Figure 1:**
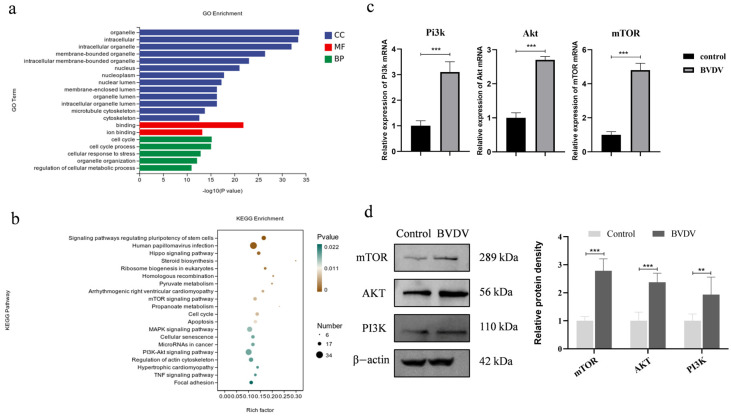
Quantitative analysis of transcriptome. (**a**) GO annotation and (**b**) KEGG pathway enrichment analysis of the DEGs in the RNA-seq transcriptomics data of BVDV-infected MDBK cells compared to mock-infected MDBK cells. (**c**) BVDV infection causes increased transcription of *Pi3k*, *Akt*, and *mTOR* (qPCR). (**d**) BVDV infection causes increased expression of PI3K, AKT, and mTOR (WB). ** *p* < 0.01, *** *p* < 0.001 (See Figures S1–S6).

To investigate the specific mechanism of TNT production after viral infection, we performed transcriptomic sequencing of virus-infected cells. The results showed that DEGs in the virus-infected group were significantly enriched in the PI3K/AKT/mTOR signaling pathway compared to the control group (Figure 1a,b). The results of both the qPCR and WB assays indicate that BVDV infection in cells could significantly increase the expression levels of PI3K, AKT, and mTOR (Figure 1c,d).

### 3.2. BVDV NS5A Significantly Induces TNT Production

**Figure 2:**
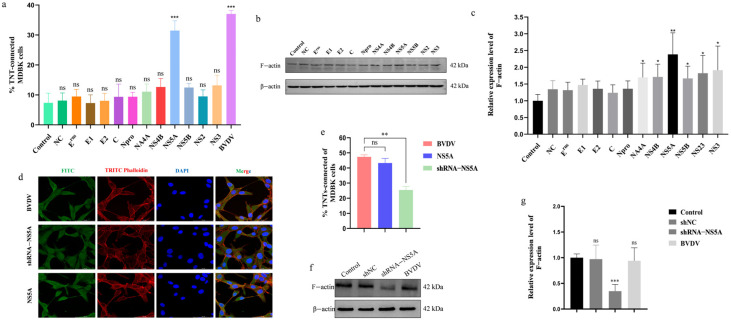
BVDV NS5A stimulates the production of TNTs. (**a**–**c**) BVDV NS5A stimulates the production of TNTs. (**d**) shNS5A reduces the number of TNTs produced, Green: FITC (BVDV); red: TRITC-phalloidin (F-actin); blue: DAPI (nuclei). (**e**) Statistical map of the number of TNTs. (**f**,**g**) shNS5A reduces F-actin expression. * *p* < 0.05, ** *p* < 0.01, *** *p* < 0.001; ns indicates not significant (p ≥ 0.05) (See Figures S1–S6).

BVDV could significantly stimulate the production of TNTs. To further investigate the specific mechanism of BVDV-induced TNT production, we lentivirally packaged all the structural and non-structural proteins of BVDV and then transfected MDBK cells. Indirect immunofluorescence results showed that only NS5A could induce a significant increase in the number of cellular TNTs produced (Figure 2a). The WB results showed that BVDV NS5A significantly stimulated the expression of F-actin, which is consistent with the indirect immunofluorescence results (Figure 2b,c).

We synthesized shRNA sequences targeting NS5A to further validate the effect of NS5A on TNT production in BVDV-infected cells. The results showed a significant reduction in the number of TNTs produced upon NS5A inhibition (Figure 2d,e). After silencing NS5A, a significant decrease in the expression level of F-actin (a major component of TNTs) was detected using WB (Figure 2f,g).

### 3.3. BVDV NS5A Significantly Induces the Formation of TNTs Between MDBK Cells

**Figure 3:**
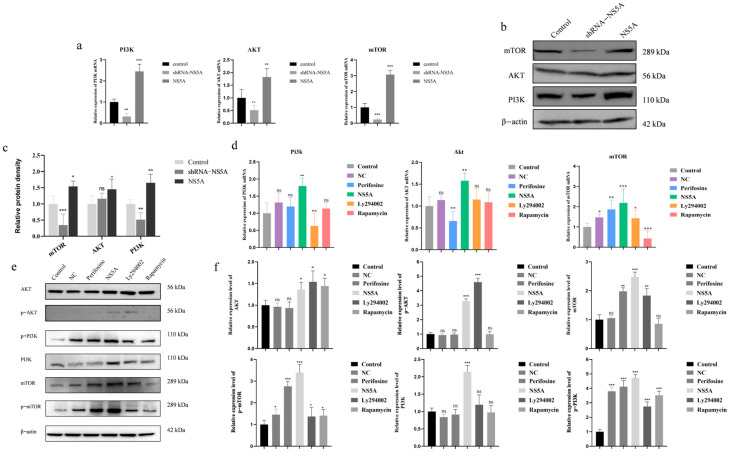
BVDV NS5A stimulates the PI3K/AKT/mTOR signaling pathway. (**a**) shNS5A reduced *Pi3k*, *Akt*, and *mTOR* transcript levels. (**b**,**c**) shNS5A reduced PI3K, AKT, and mTOR protein expression levels. (**d**) NS5A causes increased levels of *Pi3k*/*Akt*/*mTOR* transcription. (**e**,**f**) BVDV NS5A causes increased phosphorylation of PI3K, AKT, and mTOR. * *p* < 0.05, ** *p* < 0.01, *** *p* < 0.001; ns indicates not significant (*p* ≥ 0.05) (See Figures S1–S6).

Furthermore, we explored the effect of silencing NS5A on the activation of the PI3K/AKT/mTOR signaling pathway. RT-qPCR results showed that the transcript levels of *Pi3k*, *Akt*, and *mTOR* were all significantly decreased after silencing BVDV NS5A; when transfected with NS5A alone, the transcript levels of *Pi3k*, *Akt*, and *mTOR* were all significantly increased (Figure 3a). The WB results showed that when silencing BVDV NS5A, PI3K and mTOR expression decreased significantly, and there was no significant difference in the AKT expression; when transfected with NS5A alone, the protein expression levels of PI3K, AKT, and mTOR increased significantly (Figure 3b,c). The above results indicate that BVDV NS5A could activate the PI3K/AKT/mTOR signaling pathway. To further explore the regulatory role of BVDV NS5A on this signaling pathway, cells were treated with inhibitors of PI3K, AKT, and mTOR and then transfected with lentiviral particles expressing NS5A to continue cultivation. The qPCR results showed that NS5A could significantly up-regulate the mRNA transcription levels of *Pi3k*, *Akt*, and *mTOR*, and the gene expression levels were suppressed when treated with inhibitors (Figure 3d). Western blot results showed that BVDV NS5A could significantly increase the phosphorylation levels of PI3K, AKT, and mTOR, and the phosphorylation levels of the proteins were inhibited when treated with the inhibitor, indicating that BVDV NS5A could activate the PI3K/AKT/mTOR signaling pathway (Figure 3e,f).

**Figure 4:**
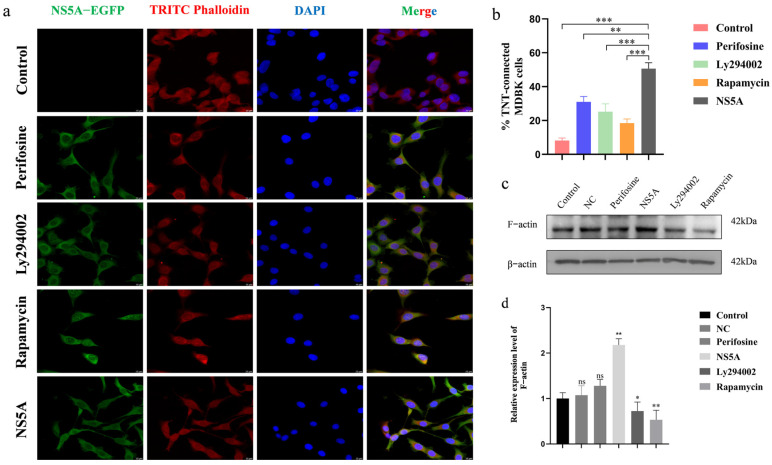
(**a**) Inhibition of the PI3K/AKT/mTOR signaling pathway reduced TNT production, Green: EGFP (NS5A); red: TRITC-phalloidin (F-actin); blue: DAPI (nuclei). (**b**) Statistical map of the number of TNTs. (**c**,**d**) BVDV NS5A can cause a significant increase in F-actin expression. * *p* < 0.05, ** *p* < 0.01, *** *p* < 0.001; ns indicates not significant (*p* ≥ 0.05) (See Figures S1–S6).

Cells were treated with PI3K, AKT, and mTOR inhibitors and then transfected with lentiviral particles expressing NS5A for further incubation to investigate the effect of inhibiting the PI3K/AKT/mTOR signaling pathway on TNT production. The results showed a significant decrease in the number of TNTs produced compared with transfection of NS5A alone (Figure 4a,b). The F-actin protein expression level was also significantly reduced (Figure 4c,d).

### 3.4. Binding of BVDV NS5A to CKAP2 Promotes the Generation of TNTs Between MDBK Cells

**Figure 5:**
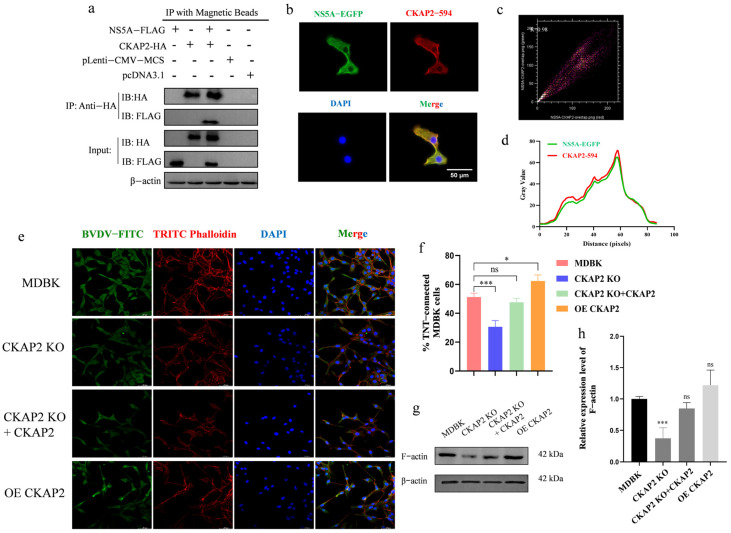
(**a**) Co-immunoprecipitation (Co-IP) results showing that CKAP2 interacts with NS5A. (**b**–**d**) Laser confocal microscopy results showing that CKAP2 interacts with NS5A, Green: EGFP (NS5A); red: Alexa Fluor 594 (CKAP2); blue: DAPI (nuclei). (**e**) Knockout of CKAP2 significantly reduces the formation of intercellular tunnel nanotubes, Green: FITC (BVDV); red: TRITC-phalloidin (F-actin); blue: DAPI (nuclei). (**f**) Statistical map of the number of TNTs. (**g**,**h**) Knockout of CKAP2 significantly reduces the protein expression levels of F-actin. * *p* < 0.05, *** *p* < 0.001; ns indicates not significant (*p* ≥ 0.05) (See Figures S1–S6).

Next, the interaction between NS5A and CKAP2 was further explored. The results showed that BVDV NS5A significantly stimulated F-actin expression, and the host protein CKAP2 was screened for NS5A interactions by CO-IP (Figure 5a); laser confocal microscopy also showed that NS5A interacts with CKAP2 (Figure 5b–d), which showed that BVDV NS5A could bind to CKAP2 and thus promote the production of intercellular TNTs in MDBK cells. To verify the role of CKAP2 in the formation of TNTs, we constructed a CKAP2-deficient cell line and observed the generation of TNTs with laser confocal microscopy. The results showed that the number of tunneling nanotubes generated in the CKAP2-deficient cell line was significantly reduced and the expression of F-actin was significantly down-regulated compared with that of the normal culture group MDBK (Figure 5e–h).

**Figure 6:**
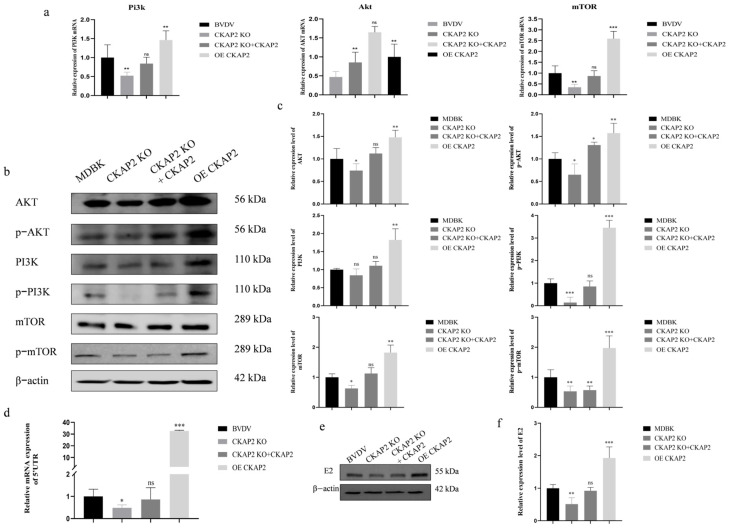
(**a**) CKAP2 causes elevated levels of *Pi3k*, *Akt*, and *mTOR* transcripts. (**b**,**c**) CKAP2 promotes phosphorylation of PI3K, AKT, and mTOR. (**d**) CKAP2 deletion leads to decreased transcript levels in BVDV 5′UTR. (**e**,**f**) CKAP2 deletion leads to a significant decrease in BVDV E2 protein expression levels. * *p* < 0.05, ** *p* < 0.01, *** *p* < 0.001; ns indicates not significant (*p* ≥ 0.05) (See Figures S1–S6).

Next, the effect of the *ckap2* gene on the PI3K/AKT/mTOR signaling pathway was further explored. The qPCR results showed that gene transcription of *Pi3k*, *Akt*, and *mTOR* was significantly decreased when the *ckap2* gene was knocked down (Figure 6a); the WB results showed that *ckap2* gene deletion caused a significant decrease in the expression and phosphorylation levels of PI3K, AKT, and mTOR (Figure 6b,c).

### 3.5. Knockdown of CKAP2 Inhibits BVDV Replication

The effect of the *ckap2* gene on BVDV replication was further explored. The results showed that the transcript level of 5′UTR was significantly decreased when the *ckap2* gene was knocked down (Figure 6d). The WB results showed that the protein expression level of BVDV E2 was significantly decreased in the MDBK CKAP2 KO culture group (Figure 6e,f). The IPMA results showed that the viral titer in the control group was 10^6.125^ TCID_50_/mL, whilst that in the knockout group was 10^5.675^ TCID_50_/mL; compared with the control group, the viral titer in the knockout group was significantly reduced.

**Figure 7:**
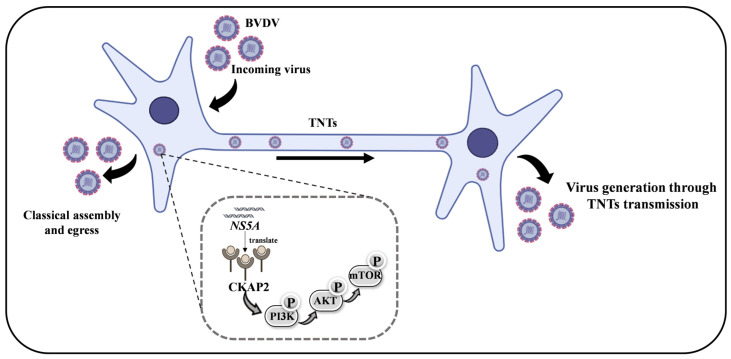
Schematic diagram of the mechanism of tunnel nanotubes as a new pathway for intercellular transmission of BVDV.

In summary, we conclude that BVDV NS5A interacts with CKAP2 to activate the PI3K/AKT/mTOR signaling pathway to promote the generation of TNTs, which facilitates BVDV transmission to promote viral replication (Figure 7).

## 4. Discussion

Bovine viral diarrhea virus (BVDV) represents a major pathogenic threat to the global cattle industry, causing substantial annual economic losses worldwide [14,15]. Recognized for its significant impact, BVDV is classified as a Category B infectious disease by the World Organization for Animal Health and is designated as a Category 2 infectious disease in China. Despite its widespread prevalence across 88 countries, the exact pathogenic mechanisms of BVDV remain incompletely elucidated. This knowledge gap underscores the critical importance of understanding BVDV transmission dynamics to effectively control its epidemiological spread.

Tunneling nanotubes constitute a recently discovered mode of intercellular communication, initially characterized by Gerdes and colleagues in 2004. These membranous channels have since been documented in multiple cell types, including murine pheochromocytoma (PC12) cells [16], human renal epithelial (HEK293T) cells [17], renal proximal tubular epithelial cells (RPTECs) [18], bladder carcinoma cells (RT4 and T24) [19], cervical adenocarcinoma cells (HeLa) [20], natural killer (NK) cells [21], human lung adenocarcinoma cells (A549) [22], and acute monocytic leukemia cells (THP-1) [23]. Notably, TNTs can also form heterotypically between distinct cell types, as demonstrated in co-culture systems involving endothelial progenitor cells (EPCs) and human umbilical vein endothelial cells (HUVECs) [24], HEK293T cells and renal fibroblasts (COS-7) [25], hematopoietic stem cells (HSCs) and macrophages [26], as well as bladder cancer cells (T24 and RT4) [27]. A notable finding in TNT research is their role in mediating intercellular pathogen transfer, including bacteria, viruses, and prions [19,28,29]. These structures appear to function as intercellular conduits for material transport. While viral transmission typically occurs through extracellular dissemination across considerable distances, emerging evidence demonstrates that tunneling nanotubes enable more efficient cell-to-cell viral spread compared to conventional extracellular transmission mechanisms. These findings suggest that direct cell-to-cell viral transmission may represent a more efficient infection mechanism than extracellular dissemination. For example, in HIV-1-infected patients, the presence of abundant TNTs between T cells provides a novel route for viral spread, enabling HIV-1 to traverse TNT surfaces and infect neighboring cells (Xu et al., 2009) [30]. Similarly, Djurkovic et al. demonstrated that the Ebola virus (EBOV) utilizes TNTs as an alternative dissemination route, proposing a novel model for intrahost EBOV spread [11]. In another study, Pepe et al. reported that TNTs facilitate SARS-CoV-2 transmission, uncovering a previously unknown mechanism that could allow viral entry into non-permissive cells and enhance infection in susceptible cell populations [12]. Moreover, viral spread via TNTs may help pathogens evade host immune defenses, thereby sustaining persistent infections. In this study, we show that bovine viral diarrhea virus (BVDV) significantly enhances TNT formation in MDBK cells, suggesting a potential strategy for BVDV to escape immune detection.

Wang Yan demonstrated the direct induction of TNT formation between astrocytes and neurons mediated by hydrogen peroxide (H_2_O_2_), revealing a defense mechanism in stressed cells. Their study indicated that the activation of the Akt/PI3K/mTOR pathway is essential for TNT formation [31]. Lin’s research further established that the formation of ROS/mtROS-induced TNTs is dependent on the PI3K/AKT/mTOR pathway and that the generation of nanomaterial-stimulated TNTs is facilitated by the activation of this pathway [32]. Our findings indicate that the PI3K/AKT/mTOR pathway was significantly enriched following BVDV infiltration of the cells. We conclude that BVDV stimulates the production of TNTs in MDBK intercellular cells through the activation of the PI3K/AKT/mTOR signaling pathway.

To further validate these results, we applied the PI3K inhibitor LY294002 and the mTOR inhibitor rapamycin, which demonstrated the important roles of PI3K and mTOR in TNT induction. Additionally, TNT development was inhibited by the AKT inhibitor perifosine, suggesting that AKT also contributes to TNT development. Notably, the expression levels of F-actin were significantly more pronounced in the LY294002 and rapamycin-treated groups compared to the perifosine-treated group. This leads us to speculate that PI3K and mTOR may play more critical roles in the generation of TNTs stimulated by BVDV. Our results also suggest that CKAP2 binds to BVDV NS5A to promote the generation of tunneling nanotubes, which contributes to the propagation of BVDV and thus viral replication. We therefore conclude that BVDV NS5A binds to CKAP2 to activate the PI3K/AKT/mTOR signaling pathway to promote the production of TNTs among MDBK cells. In addition, the experimental results suggested that CKAP2 binds to BVDV NS5A to promote the generation of tunneling nanotubes, which contributes to the spread of BVDV and thus viral replication. Our results suggested a key regulatory role of CKAP2 in TNT generation. And ckap2 gene deletion leads to a significant reduction in TNT number and a subsequent significant inhibition of viral replication efficiency.

In summary, this study confirms that BVDV infection induces the production of TNTs in MDBK cells, elucidates the molecular mechanism by which BVDV NS5A promotes viral transmission and replication via TNTs through the CKAP2-PI3K/AKT/mTOR signaling pathway, and reveals a novel pathway by which BVDV establishes an efficient intercellular transmission channel.

## Data Availability

The data generated or analyzed during this study are included in this article and its Supplementary Information file. The original sequencing data of omics reported in this paper have been deposited in the China National Center for Bioinformation/Beijing Institute of Genomics, Chinese Academy of Sciences (Shared URL: https://ngdc.cncb.ac.cn/gsa/s/9vN11252 (accessed on 6 November 2025)) and can be accessed at https://ngdc.cncb.ac.cn/gsa/ (accessed on 6 November 2025).

## Supplementary Materials

The following supporting information can be downloaded at https://www.mdpi.com/article/10.3390/vetsci13060505/s1: Figures S1–S6: Original Western blot images.

## Abbreviations

The following abbreviations are used in this manuscript:

BVDV	Bovine viral diarrhea virus
TNTs	Tunneling nanotubes
PVDF	Polyvinylidene fluoride
MDBK	Madin-Darby bovine kidney
DMEM	Dulbecco’s modified eagle’s medium
PFA	Paraformaldehyde
TBS	Tris-buffered saline
HRP	Horseradish peroxidase
ECL	Emitter-coupled logic
