# Supplementary material for: BVDV NS5A Binds to CKAP2 and Activates the PI3K/AKT/mTOR Pathway to Facilitate Virus Transmission Through Tunneling Nanotubes
Source: Vet Sci. 2026 May 22;13(6):505. doi: 10.3390/vetsci13060505 (PMC13307808; doi:10.3390/vetsci13060505)

Fig.S1.d mTOR

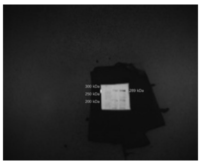

Fig.S1.d AKT

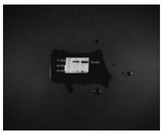

Fig.S1.d PI3K

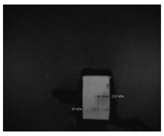

Fig.S1.d β-actin

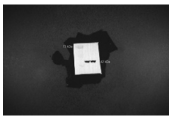

Fig.S2.b F-actin

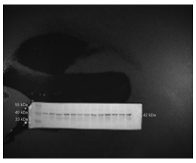

Fig.S2.b β-actin

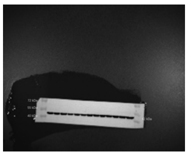

Fig.S2.f F-actin

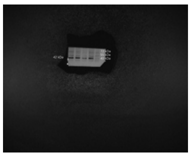

Fig.S2.f β-actin

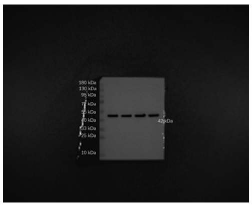

Fig.S3.b mTOR

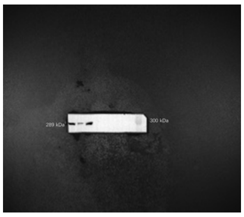

Fig.S3.b AKT

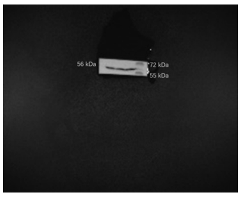

Fig.S3.b β-actin

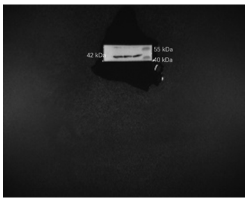

Fig.S3.e AKT

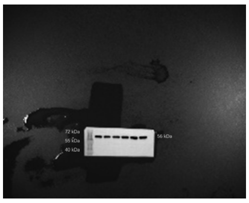

Fig.S3.e p-AKT

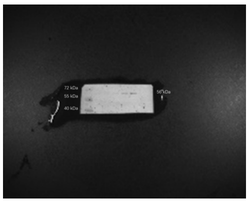

Fig.S3.e PI3K

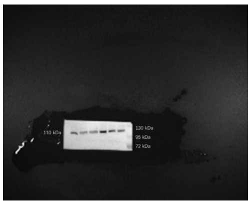

Fig.S3.e mTOR

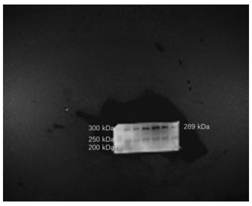

Fig.S3.e p-mTOR

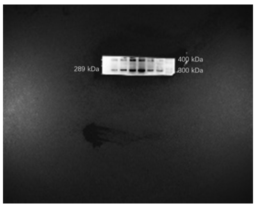

Fig.S3.e β-actin

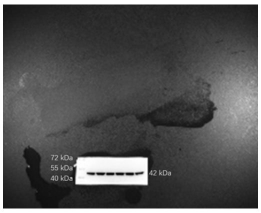

Fig.S4.c F-actin

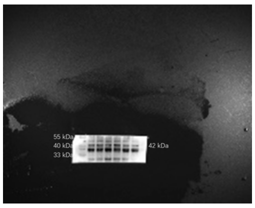

Fig.S5.g β-actin

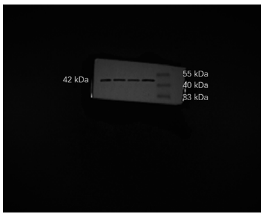

Fig.S6.b AKT

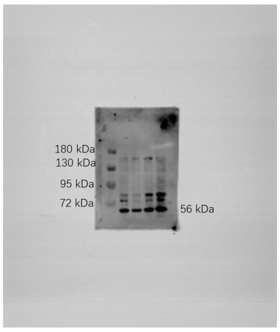

Fig.S6.b p-AKT

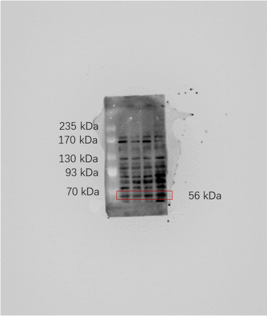

Fig.S6.b PI3K

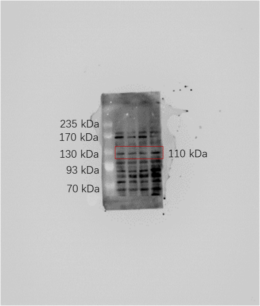

Fig.S6.b p-PI3K

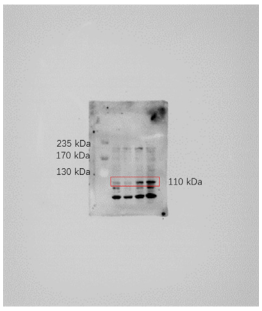

Fig.S6.b mTOR

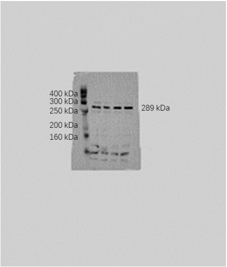

Fig.S6.b p-mTOR

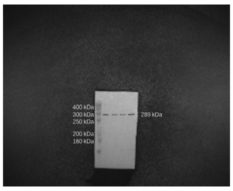

Fig.S6.b β-actin

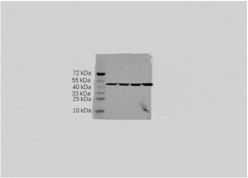

Supplement: Supplementary file 1 [file vetsci-13-00505-s001.zip › vetsci-4295486-supplementary.pdf]
